# Novel post-transcriptional dimension of glucocorticoid action through mRNA translation and P-body remodeling

**DOI:** 10.1038/s41392-026-02760-y

**Published:** 2026-06-24

**Authors:** Victoria J. Nicolini, THE FRENCH GR-P-BODY NETWORK, Mathilde Dupart, Wolfgang Raffelsberger, Olivia Vidal-Cruchez, Tifenn Rete, Karine Jacquet, Frédéric Brau, Sophie Abelanet, Marie Irondelle, Antonin Bourdin, Jérôme Durivault, Célia Gotorbe, Adeline Knittel-Obrecht, Bruno Didier, Pascal Villa, Christophe Di Giorgio, Baharia Mograbi, Arnaud Hubstenberger, Paul Hofman, Patrick Brest

**Affiliations:** 1 Université Côte d’Azur (UCA), Institute for Research on Cancer and Aging, Nice (IRCAN), INSERM U1081, CNRS UMR7284, Nice, France; 2 CHU-Nice, Laboratory of Clinical and Experimental Pathology (LPCE), Hospital-Integrated Biobank (BB-0033-00025), Nice, France; 3 IHU-RespirERA, FHU-OncoAge, Nice, France; 4 Institut de Génétique et de Biologie Moléculaire et Cellulaire (IGBMC), INSERM U1258, CNRS UMR 7104, Université de Strasbourg, Illkirch, France; 5 Robert H. Lurie Comprehensive Cancer Center, Northwestern University, Chicago, IL 60611 USA; 6 Department of Biochemistry and Molecular Genetics, Feinberg School of Medicine, Northwestern University, Chicago, IL USA; 7 Université Côte d’Azur (UCA), Institut de Biologie Valrose (iBV), Nice, France; 8 Université Côte d’Azur (UCA), Institut de Pharmacologie Moléculaire et Cellulaire (IPMC), Sophia-Antipolis, France; 9 Université Côte d’Azur (UCA), Centre Méditerranéen de Médecine Moléculaire (C3M), Nice, France; 10 Centre Scientifique de Monaco, Département de Biologie Médicale, Monaco, Monaco; 11 Plateforme de Chimie Biologique Intégrative de Strasbourg (PCBIS) UAR3286, Strasbourg, France; 12 Laboratoire d’Innovation Thérapeutique UMR7200, Strasbourg, France; 13 Université Côte d’Azur (UCA), Institut de Chimie de Nice (ICN), UMR 7272, Faculté des Sciences, Nice, France

**Keywords:** Cell biology, Molecular biology, Health care

**Dear Editor**,

Processing-bodies (P-bodies) are cytoplasmic membraneless organelles that regulate mRNA translation through phase separation. Despite extensive molecular characterization, the physiological signals controlling their assembly remain poorly understood. Sodium arsenite, a widely used P-body chemical regulator, causes rapid cell death, limiting insights into regulatory mechanisms.

Through an unbiased screen of 1520 FDA-approved drugs, we identified glucocorticoids as potent inducers of P-body formation, establishing an unrecognized connection between steroid hormone signaling and cytoplasmic RNA granule dynamics (supplementary figures deposited in Zenodo; https://zenodo.org/records/17368807). Glucocorticoids such as dexamethasone and prednisolone, widely prescribed, increased P-body numbers at clinically relevant concentrations (0.1–1 μM) within 48 h across multiple cell lines (A549, HeLa, and Mel501) (Fig. 1a left). To assess glucocorticoid receptor (GR) involvement and system dynamics, we used complementary approaches: GR antagonism (RU486) completely blocked P-body formation and FKBP5 upregulation, while glucocorticoid withdrawal rapidly reversed the phenotype within 24 h (Fig. 1a, right). This GR-dependent reversible response defines a tightly regulated pathway.

**Fig. 1:**
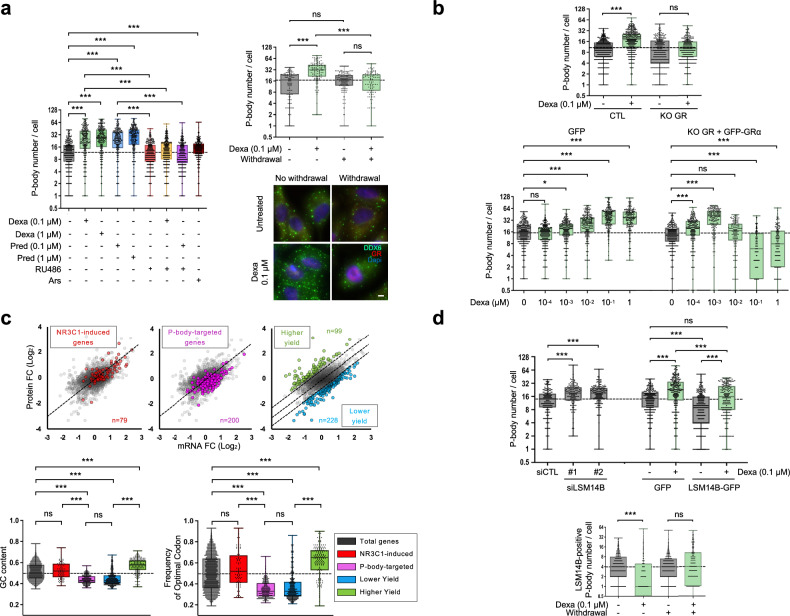
Novel post-transcriptional dimension of glucocorticoid action through mRNA translation and P-body remodeling. **a** Glucocorticoid receptor (GR) activation increases P-body numbers in different epithelial cell lines. (Left) Quantification of P-body numbers per cell in A549 cells treated for 48 h with Dexa (0.1 or 1 µM), Pred (0.1 or 1 µM), RU486 (1 µM; a glucocorticoid inhibitor, 1 h pre-treatment), or sodium arsenite (Ars; 0.5 mM for 30 min). (Right) Quantification of P-body numbers per cell in A549 cells treated with 0.1 µM Dexa for 48 h, followed by 24 h in reference medium (Withdrawal), with corresponding immunofluorescence images showing DDX6 (green), glucocorticoid receptor (GR, red), and nuclei (DAPI, blue). **b** The GRα isoform is required for glucocorticoid-induced P-body accumulation. (Top) Quantification of P-body numbers per cell in A549 control (CTL) or GR knockout (KO GR) cells treated with 0.1 µM Dexa for 48 h. (Bottom) Quantification of P-body numbers per cell in A549 GFP and A549 KO GR cells rescued with GFP-GRα, treated for 48 h with Dexa ranging from 10^−4^ to 1 µM. **c** GR activation promotes a P-body-associated AU-rich bias in mRNA translation fate. (Top) Correlation of differential mRNA (RNA-seq) and protein (mass spectrometry) fold changes after 48 h of 1 µM Dexa treatment. Highlighted gene sets include NR3C1-induced genes (red), P-body-targeted genes (purple), and genes showing increased (green) or decreased (blue) protein output relative to mRNA changes. (Bottom) GC content of CDS (coding sequence) for each gene category and the frequency of optimal codon (Fop) within CDS regions for the same gene categories. **d** LSM14B expression inversely correlates with GR-dependent P-body accumulation. (Top) Quantification of P-body numbers in A549 cells transfected with siCTL or two siRNAs against LSM14B, and in GFP and LSM14B-GFP A549 cells treated with 0.1 µM Dexa for 48 h. (Bottom) LSM14B-positive P-body numbers per cell in A549 cells treated with 0.1 µM Dexa for 48 h and followed by Dexa withdrawal for 24 h (Withdrawal). Scale bars: 10 µm. P-bodies were quantified in at least 100 cells per condition from at least three independent biological experiments. After Shapiro-Wilk normality testing, data were analyzed using Kruskal-Wallis tests with Dunn’s post hoc comparisons. Significance: * *p* < 0.033, ** *p* < 0.002, *** *p* < 0.001; ns not significant

Using loss-of-function (CRISPR/Cas9 knockout) (Fig. 1b top) and rescue approaches, we demonstrated that the glucocorticoid-responsive GRα isoform, specifically, but not GRβ or truncated variants,^1^ was both necessary and sufficient for glucocorticoid-induced P-body formation. Dose-response studies revealed an inverted U-shaped curve with GRα overexpression (Fig. 1b bottom), indicating that both insufficient and excessive GR signaling can disrupt optimal P-body assembly, suggesting precise regulation of this pathway. Importantly, glucocorticoid-induced P-bodies form independently of G3BP1-positive stress granules, distinguishing this response from classical stress responses.

Because P-body formation results from RNA repression,^2^ we investigated whether increased P-body abundance under glucocorticoid treatment reflects global or selective translational regulation. Unlike translation inhibitors such as cycloheximide, glucocorticoids did not cause a global shutdown of protein synthesis. SUnSET assay confirmed sustained protein production under dexamethasone treatment, suggesting a selective post-transcriptional response rather than a widespread protein synthesis repression. Matched transcriptomic and proteomic analyses following GR activation exhibited robust glucocorticoid gene set enrichment signatures (GSEA NES = 2.54 and 2.71, *p* < 10^−10^ respectively). However, analysis of differential mRNA-protein correlations revealed striking selectivity: canonical GR (NR3C1)-induced gene signatures were correlated, whereas P-body-targeted transcripts, previously defined in purified P-bodies,^3^ exhibited marked decorrelation, with mRNA accumulation (NES = 1.92, *p* < 10^−10^) but protein depletion (NES = − 1.84, *p* < 2.1 × 10^−6^) (Fig. 1c top). This accumulation was sequence- and codon-dependent. First, previously annotated P-body-associated transcripts exhibited an AU-rich composition across both their coding sequences and 3’UTRs, consistent with previous observations. Second, these transcripts displayed reduced Codon Adaptation Index (CAI) and Frequency of Optimal Codons (Fop)^4^ specifically within their coding sequences, two parameters directly linked to translational efficiency (Fig. 1c bottom). Third, genome-wide analysis showed that genes whose protein response to dexamethasone was attenuated relative to their mRNA induction shared these same sequence and codon features, extending this selectivity beyond annotated P-body-targeted transcripts. In contrast, GC-rich transcripts with higher CAI and Fop exhibited increased protein levels, indicating that sequence- and codon-dependent selectivity represents a general post-transcriptional dimension of glucocorticoid action.

Restricted proteomic analysis of P-body core component proteins under glucocorticoid treatment revealed LSM14B downregulation experimentally demonstrated by a significant decreased number of LSM14B-positive P-bodies. While its paralogue LSM14A is a core essential component of P-bodies (as DDX6 and 4-ET) and acts as a translational repressor,^5^ LSM14B controls oocyte mRNA storage and stability to ensure female fertility. SiRNA-mediated knockdown of LSM14B recapitulated the glucocorticoid-induced phenotype. Although dexamethasone still induced a residual increase in P-body number in LSM14B-overexpressing cells, this response was significantly attenuated compared to control cells (Fig. 1d top). Notably, the number of LSM14B-positive P-bodies mirrored the previously observed dynamic (Fig. 1a), returning to baseline number within 24 h of glucocorticoid withdrawal (Fig. 1d bottom). These converging results support a functional contribution of LSM14B to glucocorticoid-driven P-body remodeling.

To investigate the mechanism underlying glucocorticoid-induced LSM14B downregulation, we conducted a systematic analysis that revealed post-transcriptional regulation since RNA-seq showed increased mRNA levels concurrent with decreased protein expression following GRα isoform activation. In this context, we systematically excluded several regulatory pathways: GR depletion did not affect baseline LSM14B levels, ruling out direct cytoplasmic GR-mRNA interactions; LSM14A levels remained unchanged, excluding indirect regulation through its paralog; LSM14B protein is stable under basal conditions after cycloheximide treatment, demonstrating absence of post-translational regulation. Interestingly, differential responses between endogenous and exogenous LSM14B seem to implicate 3’UTR-dependent regulation. The LSM14B 3’UTR lacks annotated GR-responsive RNA-binding protein and microRNA binding sites, consistent with non-canonical regulatory interactions. Although the precise molecular mediators remain to be identified, our complementary gain- and loss-of-function experiments suggest a functional link between GR signaling and LSM14B regulation, providing a framework for future mechanistic studies.

The glucocorticoid-induced increase in P-body number is associated with an expanded pool of AU-rich, low-CAI transcripts exhibiting reduced protein output, consistent with enhanced transcript partitioning into P-bodies. This mRNA content-dependent partitioning offers a conceptual framework applicable to various stress contexts. AU-rich, low-CAI transcripts are preferentially repressed, while GC-rich, high-CAI housekeeping transcripts remain available for translation, enabling glucocorticoids to reshape cellular proteomes without direct transcriptional control. As essential stress hormones, glucocorticoids may use P-body remodeling as a conserved mechanism for proteomic adaptation. Notably, our in silico analysis of inflammatory effectors suggests that AU-rich, low-CAI transcripts include key cytokines (IL-1A, IL-8) and immune regulators (IL-15, IL-18), indicating a potential mechanistic link to glucocorticoid action that may depend on cellular and physiological context.

These findings expand our understanding of glucocorticoid action beyond the canonical transcriptional model, revealing an intersection between hormonal signaling and RNA translation fate. The dynamic and reversible nature of this post-transcriptional regulation observed in cellulo demonstrates that translational control can adjust proteome composition. While the functional consequences in vivo remain to be tested, this work provides a conceptual framework for understanding how glucocorticoids orchestrate cellular adaptation to stress and highlights a previously underappreciated layer of post-transcriptional regulation.^1^

In conclusion, this work reveals a novel post-transcriptional dimension of glucocorticoid action, expanding our understanding of how these essential stress hormones modulate proteome composition and translational control in cellular stress contexts.

## Supplementary information

SUPPLEMENTAL MATERIAL

## Data Availability

All additional figures that could not be included in the main figure due to the Letter format requirements have been deposited in Zenodo and publicly available at the following https://zenodo.org/records/17368807. Whole transcriptomic data were similarly deposited under accession number GSE295999 and the proteomic data accession number Pride PXD063431.

## References

[1] Ramamoorthy, S. & Cidlowski, J. A. Corticosteroids-mechanisms of action in health and disease. *Rheum. Dis. Clin. North Am.* **42**, 15–31 (2016). 26611548 10.1016/j.rdc.2015.08.002 PMC4662771

[2] Eulalio, A., Behm-Ansmant, I., Schweizer, D. & Izaurralde, E. P-body formation is a consequence, not the cause, of RNA-mediated gene silencing. *Mol. Cell. Biol.* **27**, 3970–3981 (2007). 17403906 10.1128/MCB.00128-07 PMC1900022

[3] Hubstenberger, A. et al. P-body purification reveals the condensation of repressed mRNA regulons. *Mol. Cell* **68**, 144–157.e5 (2017). 28965817 10.1016/j.molcel.2017.09.003

[4] Subramanian, K., Payne, B., Feyertag, F. & Alvarez-Ponce, D. The codon statistics database: a database of codon usage bias. *Mol. Biol. Evol.* **39**, msac157 (2022). 35859338 10.1093/molbev/msac157 PMC9372565

[5] Ayache, J. et al. P-body assembly requires DDX6 repression complexes rather than decay or Ataxin2/2 L complexes. *Mol. Biol. Cell* **26**, 2579–2595 (2015). 25995375 10.1091/mbc.E15-03-0136 PMC4501357

