# Supplementary material for: Novel post-transcriptional dimension of glucocorticoid action through mRNA translation and P-body remodeling
Source: Signal Transduct Target Ther. 2026 Jun 24;11:245. doi: 10.1038/s41392-026-02760-y (PMC13291245; doi:10.1038/s41392-026-02760-y)
Supplement: Supplementary file 1 — SUPPLEMENTAL MATERIAL [file 41392_2026_2760_MOESM1_ESM.docx]

Supplementary Materials for

Novel post-transcriptional dimension of glucocorticoid action through mRNA translation and P-body remodeling

Victoria J. Nicolini, The French Gr-P-Body Network, Patrick Brest

Correspondence to: [patrick.brest@univ-cotedazur.fr](mailto:patrick.brest@univ-cotedazur.fr)

**This PDF file includes:**

- Materials and Methods

Materials and Methods

Cell culture

A549 (human lung adenocarcinoma epithelial cell line, LUAD, ATCC Cat# CCL-185, RRID: CVCL_0023), HeLa (human uterus; cervix adenocarcinoma epithelial cell line, ATCC Cat# CCL-2, RRID: CVCL_0030), and Mel501 (melanoma epithelial cell line, SKCM, RRID: CVCL_4633, kind gift from Dr. Gillot Laboratory) cells were cultured according to ATCC recommendations in DMEM (A549, HeLa) or RPMI 1640 (Mel501) supplemented with 5% fetal bovine serum at 37°C in 5% CO₂. Cells were used for less than one month, authenticated by STR profiling (Eurofins Genomics), and routinely tested for mycoplasma (Plasmotest, Invivogen).

Treatments

Cells were treated with TGFβ (Sigma, Cat# SRP3171, 10 ng/ml, 72 h), dexamethasone (Bertin Bioreagent, Cat# 20340-100), prednisolone (Bertin Bioreagent, Cat# 15933-1), RU486 (Cayman Chemical, Cat# 10006317, 1 µM, 1 h pre-treatment), sodium arsenite (Sigma, Cat# 1.06771000, 0.5 mM, 30 min), cycloheximide (Sigma, Cat# C6255, 30 µg/ml, 1 h) or puromycin (Invivogen, Cat# ant-pr-1, 10 µg/ml, 10 min).

For siRNA experiments, 200,000 cells were seeded in 6-well plates and transfected after 24 h with control siRNA (Thermo Fisher, Cat# 4390844) or LSM14B siRNAs (s229767, s45634, Thermo Fisher) using JetPrime (PolyPlus). Cells were harvested 48 h post-transfection.

Drug screen

A high-content screen was performed at PCBIS (Plateforme de Chimie Biologique Intégrative de Strasbourg) using the Prestwick Chemical Library (1,520 FDA-approved compounds). A549 cells stably expressing DDX6-GFP were seeded in 96-well plates and treated with compounds (10 µM, 72 h; 0.1% DMSO final). Images were acquired every 4 h using the IncuCyte-S3 system and analyzed for confluency and P-body number per cell. Compounds were selected based on ≥65% confluency at 72 h and P-body induction exceeding TGFβ. 97 promising hits (6.4%) were then subjected to secondary validation assays at both 1 µM and 10 µM to confirm their effects and assess dose dependency. Final results are presented in ^repository^Figure 1.

CRISPR-Cas9 gene editing

A549 wildtype (WT) cells were transfected using jetPEI (Polyplus, 101000053) according to the manufacturer's instructions with the pSpCas9(BB)-2A-GFP (PX458) plasmid (a gift from Feng Zhang; Addgene plasmid #48138; RRID: Addgene_48138) containing clustered regularly interspaced short palindromic repeats (CRISPR)-CRISPR-associated protein 9 (Cas9) targeting the following regions: exon 2 (5’-GTAGAAAAAACTGTTCGACCA-3’), exon 3 (5’-GAGCTCCTCAACAGCAACAAC-3’), and exon 5 (5’-GAACCTCCAACAGTGACACCA-3’ and 5’-GCGCTCAACATGTTAGGAGGG-3’) targeting the NR3C1 gene. GFP-positive cells were single-cell sorted (BD FACSMelody) 24 h post-transfection. Clones were screened by Western blot and validated by Sanger sequencing (Eurofins Genomics). Two independent NR3C1 knockout clones were used.

Plasmid constructs and generation of stable cell lines

The pPRIPu GFP-DDX6 plasmid used in this study was constructed as follows: the pPRIPu CrUCCI vector (kind gift from Dr. Delaunay) was amplified with primer adaptors containing AgeI and BamHI restriction sites. pEGFP-C1_p54cp plasmid (kind gift from Drs. Weil and Kress) was digested by AgeI and BamHI, and the obtained GFP-DDX6 fragment was inserted into pPRIPU after digestion with AgeI and BamHI. The pPRIPu GFP-GR plasmids used in this study were constructed as follows: the eGFP-NR3C1 (GRa) insert was digested with AgeI and BamHI from pEGFP GR (Addgene Plasmid #47504) and inserted into pPRIPU after digestion with AgeI and BamHI. GRb, GRDEx9, and GRa-RK491AA isoforms were generated from pPRIPu GFP-GRa by reverse PCR using specific primers (GRb_Fwd: 5’-AGCACATCTCACACATTAATCTGAGG- ATCCACCGGATCTAGATAACTG-3’; GRb_Rev: 5’-TTCTGGTTTTAACCACATAACATT-TTCATGCATAGAATCCAAGAGTTTTGTCAG-3’; GRDEx9_Fwd: 5’-TGAGGATCCACCG-GATCTAGATAACTG-3’; GRDEx9_Rev: 5’-TTCATGCATAGAATCCAAGAGTTTTGTCA-G-3’). The pPRIPu GFP-LSM14B plasmid used in this study was constructed as follows: LSM14B cDNA was generated using a 5’-CTTTGGCTGCACCCTCACAC-3’ primer using SuperscriptIV enzyme (Invitrogen). Then the LSM14B cDNA was amplified with primer adaptors containing XhoI and BglII restriction sites (LSM14B_Fwd: 5’-GCCCCGGTAGATCTCGG-GCCCGCGGTACCGTCGAGTCACACCCTGCCAGTCCC-3’ and LSM14B_Rev: 5’-CAGAT-CTCGAGCTCAAGCTTCGAATTCCATGAGCGGCTCCTCAGG-3’) and inserted into pPRIPu GFP-DDX6 previously digested by XhoI and BamHI (compatible with BglII). For all plasmids, the integrity of the entire sequence was confirmed by sequencing (Eurofins Genomics, Snapgene).

Replication-defective retroviral vectors were used to generate stable A549 lines. Cells were selected with puromycin (5 µg/ml, ≥48 h) and sorted for homogeneous GFP expression (FACS Aria).

Western blot

Proteins were extracted in Laemmli buffer (12.5 mM Na₂HPO₄, 15% glycerol, 3% SDS). Protein concentration was determined using DC Protein Assay (Bio-Rad). Equal amounts were separated on 15% SDS-PAGE and transferred to PVDF membranes. Membranes were blocked in 3% BSA (PBS-0.1% Tween-20) and incubated overnight at 4°C with anti-puromycin (Millipore, Cat# MABE343, RRID: AB_2566826, 1:10,000) or anti-α-actinin (Millipore, Cat# 05-384, RRID: AB_11212399, 1:10,000). HRP-conjugated secondary antibody (Promega, Cat# W4021, RRID: AB_430834, 1:5,000) was applied for 1 h. Detection was performed using ECL (Merck Millipore) and Syngene Pxi4. Quantification was done with Fiji (RRID: SCR_002285).

Immunofluorescence, microscopy, and P-body quantification

Cells were fixed in 4% paraformaldehyde (15 min, 37°C), permeabilized (0.3% Triton X-100, 5 min) and blocked (0.03% Triton X-100, 0.2% gelatin, 1% BSA). Primary antibodies (1:200, overnight, 4°C) were anti-DDX6 (Novus, Cat# NB200-191, RRID: AB_523228), anti-LSM14A (Santa Cruz, Cat# sc-398552, RRID: AB_3099527), anti-LSM14B (Thermo Fisher, Cat# PA5-66371, RRID: AB_2664653) and anti-GR (Santa Cruz, Cat# sc-393232, RRID: AB_2687823). Alexa Fluor secondary antibodies (Thermo Fisher, 1:500) were applied for 1 h. Nuclei were stained with DAPI (1:10,000). Z-stacks were acquired on a Zeiss upright microscope with a 40x/1.3 objective and ORCA-Fusion BT camera. Maximum projections were analyzed using CellProfiler v2.0 to segment cells and quantify P-bodies per cell by using the "IdentifyPrimaryObjects" function and counted per cell using the "MeasureObjectSize-Shape" and "RelateObjects" functions.

Statistical analysis for microscopy analyses

Quantitative data were described and presented graphically as boxplots with medians and standard deviations (immunofluorescence quantification). All statistical analyses were performed using Prism 10 (GraphPad, RRID: SCR_002798). Normality of the distribution was tested with Shapiro’s test. Statistical comparisons were performed using Dunn’s multiple comparison test and considered significant with an alpha level of p < 0.05 (graphically: * for p < 0.033, ** for p < 0.002, *** for p < 0.001, and ns for non-significant).

Bulk RNA-seq library preparation, sequencing, and analysis

Total RNA was extracted from A549 cells treated with or without 1 µM dexamethasone for 48h (n=4) with TRIzol reagent (Thermo Fisher) and purified on Direct-zol RNA miniprep kit (Zymo Research, Orange, CA). RNA integrity was assessed using an Agilent 2100 Bioanalyzer (RIN ≥8.5). Libraries were prepared by Novogene using 200 ng of total RNA, following ribosomal RNA depletion (Ribo Zero Gold rRNA Removal Kit, Illumina). RNA Fragmentation was carried out using divalent cations under elevated temperature in First Strand Synthesis Reaction Buffer (5X). First-strand cDNA was synthesized using random hexamer primers and M-MuLV Reverse Transcriptase (RNase H-). Second-strand cDNA synthesis was subsequently performed using DNA Polymerase I and RNase H. Remaining overhangs were converted into blunt ends via exonuclease/polymerase activities. After adenylation of the 3' ends of DNA fragments, adapters with hairpin loop structure were ligated to prepare for hybridization. To select cDNA fragments of preferentially 370-420 bp in length, the library fragments were purified with the AMPure XP system (Beverly, USA). Enzyme (3 μL) was used with size-selected, adaptor-ligated cDNA at 37°C for 15 min, followed by 5 min at 95°C before PCR. PCR was performed using Phusion High-Fidelity DNA polymerase, Universal PCR primers, and Index (X) Primer. PCR products were purified (AMPure XP system), and library quality was assessed using the Agilent 5400 system and quantified by qPCR.

Equimolar libraries were sequenced on an Illumina NovaSeq 6000 (150 bp paired-end, 40 to 50 million read pairs per sample). Reads were assessed with FastQC v0.11.9, trimmed with Cutadapt v3.4, and aligned to GRCh38 using STAR v2.7.9a. Gene counts were generated with featureCounts (Subread v2.0.3). Differential expression was performed in DESeq2 v1.32.0 via Phantasus. Genes with fold change ≥ 1.5 and adjusted p < 0.01 were considered significant. Pathway enrichment was conducted using GSEA v4.1.0 with ShinyGO (Ge et al., 2019, Bioinformatics).

Mass spectrometry analysis.

Proteomics was performed on A549 cells treated ± 1 µM dexamethasone for 48 h (n=4 biological replicates), processed in parallel with RNA-seq samples to ensure paired transcriptomic and proteomic comparison. Proteins were extracted in Laemmli buffer. For digestion, 30 µg protein was TCA-precipitated overnight at 4°C, washed twice with cold acetone, resuspended in 1 M urea (0.1 mM Tris-HCl pH 8.5), reduced (10 mM DTT, 30 min, 56°C), alkylated (20 mM iodoacetamide, 30 min, 25°C), and digested with trypsin (two additions, overnight, 37°C). Samples were acidified with 0.2% TFA. NanoLC-MS/MS was performed using an Ultimate 3000 nano-RSLC system coupled to an Exploris 480 quadrupole-orbitrap via a nano-electrospray source with FAIMS pro interface (Thermo Fisher Scientific). Tryptic peptides (1 μl) were preconcentrated on a C18 PepMap100 trap column (300 μm × 1 mm) for 1 min at 15 μL/min with 2% ACN and 0.1% FA in water. Peptide separation was achieved on an analytical column (C18 PepMap, 75 μm ID × 15 cm) using a 40-min gradient from 8% to 25% buffer B (buffer A: 0.1% FA in water; buffer B: 0.1% FA in 80% ACN) at 450 nl/min and 45°C. The gradient was followed by a regeneration step at 90% B and re-equilibration to 8% B, with a total chromatography time of 120 min. The mass spectrometer operated in positive ionization mode using Data-Dependent Acquisition with two cycles of FAIMS compensation voltages (-45V and -55V for 1.2 and 0.8 sec, respectively). Each FAIMS-DDA cycle consisted of a survey scan (350-1200 m/z, 60,000 FWHM) followed by MS² spectra acquisition (HCD; 30% normalized energy; 2 m/z window; 22,500 FWHM). Normalized AGC values were set to 300% for MS1 and 100% for MS2, with a maximum injection time of 50 ms in both scan modes. Single-charged and unassigned states were excluded, with a 40 s exclusion duration (± 10 ppm mass width).

MS data processing.

Proteins were identified using Proteome Discoverer 2.5 software (Thermo Scientific) against the Human proteome database (SwissProt, reviewed, March 2024 release). Precursor and fragment mass tolerances were set at 7 ppm and 0.05 Da, respectively, allowing up to two missed cleavages. Oxidation (M) was set as a variable modification and carbamidomethylation (C) as a fixed modification, with peptide filtering at 1% FDR. Protein quantification required at least one unique peptide, as determined by XIC values. The mass spectrometry proteomics data have been deposited to the ProteomeXchange Consortium via the PRIDE partner repository with the dataset identifier PXD063431. Subsequent bioinformatic analysis was performed in R using the 'wrProteo' (https://CRAN.R-project.org/package=wrProteo) package, including data filtering (removing proteins with ≤3 PSM or detected in <70% of samples), variance stabilization, and missing value imputation using the k-nearest neighbor method (wrProteo). Differential protein expression between dexamethasone-treated and untreated A549 cells was determined using linear models with empirical Bayes statistics. Proteins with absolute log₂ fold change ≥1.2 and adjusted p-value ≤0.05 (Benjamini-Hochberg correction) were considered significantly differentially expressed. For correlation analysis, genes with corresponding Ensembl identifiers were filtered. From these genes, we generated lists of fold change values for transcripts and proteins following dexamethasone treatment, which were plotted on the same graph to assess the correlation between mRNA and protein level changes 48 hours post-treatment.

GC content and codon usage bias analyses.

GC content was extracted from Ensembl identifiers for cDNA, 5’UTR, coding region (CDS), and 3’UTR. Codon usage bias analyses were restricted to CDS regions and quantified using three complementary metrics: Codon Adaptation Index (CAI), frequency of optimal codons (Fop), and GC content at the third codon position (GC3). Codon usage data were obtained from the Codon Statistics Database (CodonStatsDB; <http://codonstatsdb.unr.edu/>) using Homo sapiens as the reference, combining nuclear, ribosomal, and mitochondrial genes. CAI was computed as the geometric mean of the relative adaptiveness values of synonymous codons, defined from reference codon frequencies and normalized to the most frequently used codon for each amino acid. Fop was calculated as the proportion of optimal codons among all synonymous codons in the CDS, excluding methionine and tryptophan. GC3 was calculated as the fraction of guanine and cytosine nucleotides at the third position of codons within the CDS.
